# Production and perception rules underlying visual patterns: effects of symmetry and hierarchy

**DOI:** 10.1098/rstb.2012.0098

**Published:** 2012-07-19

**Authors:** Gesche Westphal-Fitch, Ludwig Huber, Juan Carlos Gómez, W. Tecumseh Fitch

**Affiliations:** 1 Department of Cognitive Biology, University of Vienna, Althanstrasse 14, 1090Vienna, Austria; 2 Messerli Research Institute, University of Veterinary Medicine Vienna, Medical University of Vienna and University of Vienna, Veterinärplatz 1, 1210 Vienna, Austria; 3 School of Psychology, St Mary's College, University of St Andrews, South Street, St Andrews, KY16 9JP, UK

**Keywords:** symmetry, plane patterns, hierarchy, pattern perception, pigeons, autism spectrum disorder

## Abstract

Formal language theory has been extended to two-dimensional patterns, but little is known about two-dimensional pattern perception. We first examined spontaneous two-dimensional visual pattern production by humans, gathered using a novel touch screen approach. Both spontaneous creative production and subsequent aesthetic ratings show that humans prefer ordered, symmetrical patterns over random patterns. We then further explored pattern-parsing abilities in different human groups, and compared them with pigeons. We generated visual plane patterns based on rules varying in complexity. All human groups tested, including children and individuals diagnosed with autism spectrum disorder (ASD), were able to detect violations of all production rules tested. Our ASD participants detected pattern violations with the same speed and accuracy as matched controls. Children's ability to detect violations of a relatively complex rotational rule correlated with age, whereas their ability to detect violations of a simple translational rule did not. By contrast, even with extensive training, pigeons were unable to detect orientation-based structural violations, suggesting that, unlike humans, they did not learn the underlying structural rules. Visual two-dimensional patterns offer a promising new formally-grounded way to investigate pattern production and perception in general, widely applicable across species and age groups.

## 1. Introduction

Abstract, non-representational visual patterns, such as those used in weaving, patchwork, embroidery, jewellery, etc., are produced in most, if not all, human cultures. Similar to music and language, such patterns seem to be a human cultural universal, not found in other species. The earliest artefacts with abstract geometrical patterns, found at Blombos cave in South Africa, predate representational art considerably [1], and archaeological findings at Avdeevo in Russia show that a Palaeolithic culture that developed representational art continued to use geometrical patterns to embellish tools and jewellery, as do modern cultures [2]. As already pointed out by Franz Boas [3] in 1927, the presence of symmetry in art is not limited to any particular culture or region.

Geometrical patterns as we understand them here are characterized by structured repetitions of elements in a two-dimensional plane. While the symmetries in patterns can be easily classified [4], the principles underlying the perception and production of geometrical patterns remain poorly studied. As Gombrich [5] noted in his classic book ‘*The sense of order*’, it is precisely because of the predictability and regularity of patterns, and also their pervasiveness in everyday culture that they are an ‘unregarded art’, often derogated to the ‘lower arts’ or ‘crafts’. Nonetheless, humans clearly like to surround themselves with visual patterns that follow some kind of structural order.

Visual patterns have in common with music and language that they are governed by a set of combinatorial principles—‘grammars’—that constrain the arrangement of units into groups on multiple hierarchical levels. Although formal language theory is most typically used in the context of linear sequences or strings (e.g. in linguistics, computer programming and molecular biology), it can be naturally extended to cover two-dimensional patterns as well [6]. These lesser-known two-dimensional extensions of formal language theory include picture grammars [7] and picture languages [8] as well as L-systems [9] and picture-processing grammars [10]. Two-dimensional variants of both regular grammars and context-free grammars are reasonably well understood [7,11], and such grammars have applications as tools for image processing [9] and as models of plant development [12]. Current research is focused on two-dimensional patterns at the finite-state level [7]. However, two-dimensional artificial grammars, and the patterns that they generate, have received little attention from psychologists interested in the production, perception and appreciation of visual patterns.

The research presented here provides a first look into this potentially rich domain, by testing various aspects of two-dimensional pattern perception, allowing people to generate their own two-dimensional patterns and analysing the output, and by comparing human two-dimensional pattern perception with that of pigeons—a highly visual bird species.

We suggest that the methods of artificial grammar learning can be fruitfully applied to the perception of visual patterns, using patterns to probe perception of structure of different sorts, preferences for different levels of structural complexity and effects of the presence or absence of hierarchy. By studying which structural manipulations make a pattern easily detected, we hope to shed light on what kind of (unconscious) knowledge a perceiver acquires concerning the regularities underlying the pattern. We can thus think of geometrical patterns as reflecting naturally occurring visual grammars. Artificial grammars based on similar principles can then be used to empirically evaluate structural parsing abilities in the visual domain.

## 2. Visual pattern production and aesthetics

One feature that sets everyday geometrical patterns apart from conventional ‘high’ art is that they can be appreciated equally by everyone, regardless of levels of artistic proficiency, cultural background or education. Similarly, the production of such patterns requires no formal art education or special artistic talents. Hence, we will first investigate what kind of patterns normal humans spontaneously create, without instructions or time constraints, reviving a neglected branch of a research programme in aesthetics outlined in 1876 by Fechner [13]. Fechner advocated that, in addition to gathering preference ratings, psychologists should also investigate material created by subjects in a controlled laboratory setting (‘Method of production: one lets many people create by themselves that which is pleasing to them’; our translation, p. 190).

While considerable research has explored the perception of symmetry and the detection of deviations from symmetry—especially bilateral symmetry—by humans and some animal species [14–18], little research has been performed to explore what kinds of symmetry and order humans produce spontaneously. Producing unstructured outputs may be difficult for humans: participants instructed to create random number sequences nonetheless produce structured sequences that deviate significantly from true randomness [19–22]. But what structures are favoured? The types of regular pattern types that are produced most, and the cultural or structural factors that determine this, remain little studied. In one pioneering study which found that humans tend to produce symmetrical patterns rather than asymmetrical patterns, participants were probably biased towards symmetry because they were instructed to produce ‘pleasing patterns’ [23]. In experiment 1, we will present a pattern production study where no such instruction biasing was present, but which still provides very similar results.

## 3. Pattern perception and gestalt principles

Gestalt psychologists searching for factors that affected the ‘goodness’ of a form [24,25] noted the influence of symmetry on figure/ground relations: a symmetrical shape is more likely to be interpreted as a figure than as background. However, these grouping principles were not explicitly formalized at the time. The concept of figural goodness was revitalized in the 1950s, in attempts to combine the intuitive understanding of symmetry with information theory [26,27]. Current Gestalt research focusing on perceptual grouping mainly explores the effects of proximity and similarity on perceptual grouping in artificial Gabor lattices, and not the more typical, everyday patterns of the type we investigate here [28,29].

We conducted several perceptual tasks to explore the perception of order in two-dimensional patterns. The main goal of our experiments was to determine which structural features help or hinder the perception of the regularity in patterns. In particular, we looked at the effects of hierarchy and symmetry within the patterns and pattern elements in discrimination efficacy. We also contrasted two patterns that differed only in one aspect of their production rule: the presence or absence of an intermediate level of structural hierarchy. We initially examined normal adults, to establish baseline values (experiment 1), and went on to test individuals with autism spectrum disorder (ASD), and children aged 5–12. ASD individuals often outperform control groups in visual search tasks, both in speed and accuracy [30–33], and there is some evidence that they give local information priority over global information when processing complex visual stimuli [34]. We explored this possibility by comparing their performance with patterns that required processing either global or local relations.

Finally, to lay the groundwork for a comparative investigation of the human ‘sense of order’, we tested whether pigeons are able to process visual patterns such as those used in our human experiments, comprising either colour or orientation features. Pigeons are interesting in this context because they are able to discriminate regular, repetitive patterns such as stripes and checkerboards from random visual patterns made of the same basic elements [35]. Although pigeons are thus able to differentiate randomness from order, it remains unclear whether they can detect minor *violations* of regular orderings and if so, which violations are most readily detectable. Pigeons are known to have a bias towards local featural processing (as opposed to global, relational processing) and we thus compared human and pigeon processing on a uniform translational pattern and a hierarchical grouped pattern—a local parsing style would enable flaw detection in the translational pattern, but not in the grouped pattern. This experiment thus begins to investigate the degree to which the perceptual mechanisms humans employ in processing abstract visual patterns are shared with other species.

## 4. Experiment 1. spontaneous pattern production

### (a) Introduction

The aim of our first experiment was to investigate the types of patterns humans spontaneously produce, when free to change the array as much or as little as they like, with no further instructions. We also investigated the effect of repeated exposure to a single particular pattern element, to see whether familiarity (or boredom) sparks creativity, by presenting each array three times in succession to each participant.

### (b) Participants

We recruited 10 adult participants (seven female, mean age: 29.3, age range: 18–51) at the University of Vienna. All participants were right-handed. None of the participants were artists or worked in creative professions. All gave their written informed consent prior to participating and were paid for participating.

### (c) Material and methods

Patterns consisted of identical ‘tiles’ arranged on a square grid. Tiles were semi-realistic depictions of actual artisanal tiles from Havana and Barcelona [36,37]. Three tile categories were used: (i) either possessing symmetry along one of the diagonals, (ii) symmetry along either the vertical or horizontal axis and/or (iii) with no internal symmetry. We used four tiles of each ‘symmetry’ type, yielding 12 tiles, each repeated three times.

**Figure 1.**
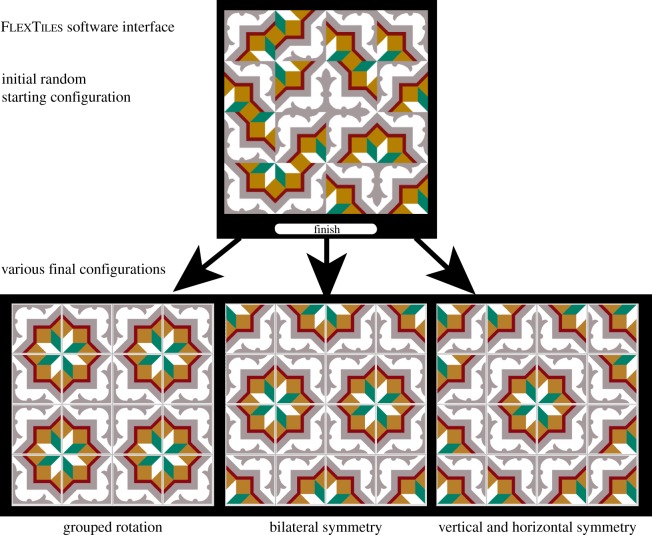
Overview of FlexTiles software interface with initial random configuration and various ordered final configurations. Tile image taken from ‘*Havana tile designs*’ published by The Pepin Press.

Using FlexTiles, a custom-written image manipulation program, each tile was repeated 36 times on a 6 × 6 matrix; initially, each tile (100 × 100 pixels) was randomly assigned to one of four possible orientations (0°, 90°, 180° or 270°). The matrices were displayed on a touch screen (Elo Intellitouch 17*″*). Every time a tile was touched, the tile rotated 90° clockwise. Participants were told that they could change the array as much or as little as they liked, and that there were no right or wrong choices. To finish their activities for each particular array (one ‘production trial’), participants touched a button on the screen below the array labelled ‘Finish’. See figure 1 for an overview of the software, in this case using tiles with diagonal symmetry. Each of the 12 individual tiles was shown in the array three times in succession. The starting arrays were always newly randomized and varied between trials. The order in which each tile was shown was also randomized. There were 36 production trials in total, split into two sessions. All instructions were given to the participants in writing and did not contain any words that alluded to beauty, aesthetics, patterns or symmetry.

In addition to the production task, each participant completed a rating task. After 18 trials, and again after all 36 production trials were completed, the participants rated single pattern arrays on a Likert scale from one to seven (1, like; 7, dislike). Rating sessions either included participants’ own versus random arrays (six of each), or random arrays versus patterns made by other humans (six each). Session order was counterbalanced across subjects, and image order within sessions was randomized. Regardless of provenance, two examples of each of the three tile symmetry classes were shown.

### (d) Results

**Figure 2.**
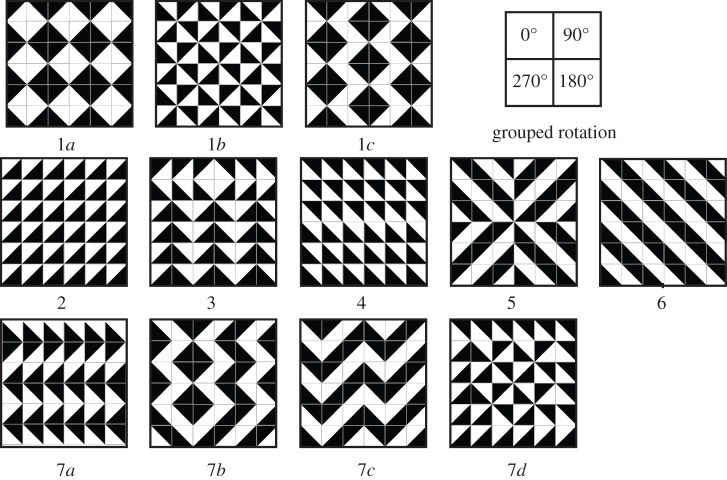
Pattern types spontaneously produced in experiment 1, recreated in black and white: (1) grouped rotation with diamond figure (1*a*), windmill figure (1*b*) or offset diamonds (1*c*). (2) Translational symmetry, (3) bilateral symmetry along vertical axis, (4) 180° rotational symmetry, (5) 180° and 90° rotational symmetry, (6) symmetry along diagonal axis. (7) examples of ‘local linear’ groupings of two tiles, with no overall global symmetry.

All participants spent a considerable amount of time spontaneously ordering the random tile arrays; the typical duration of the experiment was an hour and on average, participants clicked 68 times in the array before submitting it as ‘finished’. The majority of patterns (72%) submitted contained at least one type of symmetry, whereas 28% did not, or only incomplete symmetry. If a pattern deviated from symmetry by more than one tile, then we did not classify it as symmetrical. The ‘non-symmetrical’ patterns nonetheless often had a high degree of order as the examples in figure 2(7*a*–*d*) show, so these measures are conservative.

One order was particularly common (shown in figure 2, top row), which we call ‘grouped rotation’: this is a special instance of symmetry along the horizontal and vertical axes. This pattern is hierarchical and has an intermediate level of organization above that of the tile element: four tiles are grouped to create a diamond-shaped figure (1*a*), a windmill-shaped (1*b*) or offset diamonds (1*c*). By contrast, translational patterns (figure 2(2), though symmetrical, have no intermediate level of organization.

We developed Python-based software to automatically compute entropy [38] and symmetry values for the patterns. Entropy values of the human-produced patterns were significantly lower than those of randomly produced patterns (human mean: 1.58, random mean: 1.94 Mann–Whitney *U*-test: *p* = 0.038, *Z* = −2.07). The maximum entropy possible in our rotational framework is 2.0 (when each of the four orientations occurs equally often in the matrix), and a pattern with full translational symmetry (only one tile orientation present) has the lowest possible entropy of 0. An analysis of entropy values however does not reveal hierarchically ordered structure—image 1*a* from figure 2 also has the maximum entropy value 2.0, because all orientations occur with equal probability. Grouped rotation is thus indistinguishable from randomness by this measure.

We thus extended our code to analyse specific symmetries in the patterns, automatically detecting the following symmetry categories (figure 2): translation, grouped rotation, diagonal, 180° rotation (including 180° + 90° rotation), horizontal and vertical symmetry (H + V). Additionally, we manually included patterns that contained only one mistake (10 images, 2.78% of the data), as well as patterns that contained local regularities but not consistent global groupings (‘local linear’; e.g. images 7*a*–*d*).

**Figure 3.**
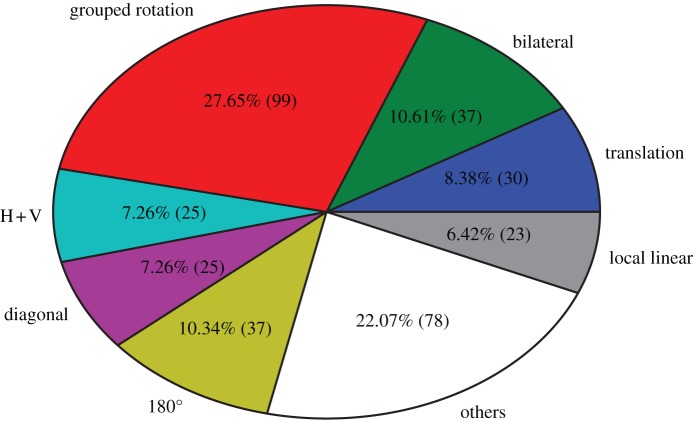
Percentages of patterns produced, with maximally one error in the patterns.

**Figure 4.**
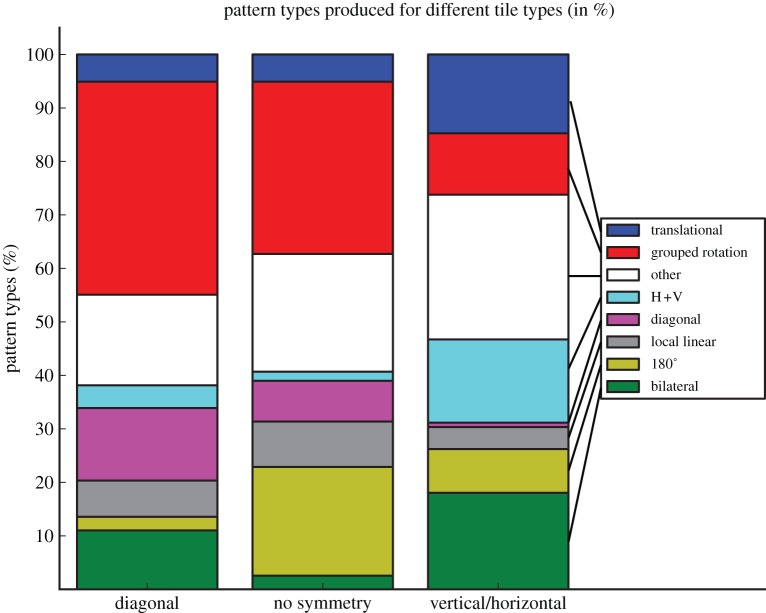
Frequencies of patterns produced for different tile types.

The only type of symmetry that was produced by every participant was ‘grouped rotation’ and was the most frequent pattern overall (figure 3). Grouped rotations made with tiles from the vertical/horizontal category were rare (11.6%), but the most frequent symmetrical pattern for tiles with diagonal symmetry (39.2%) or those containing no symmetry (31.6%; figure 4).

**Figure 5.**
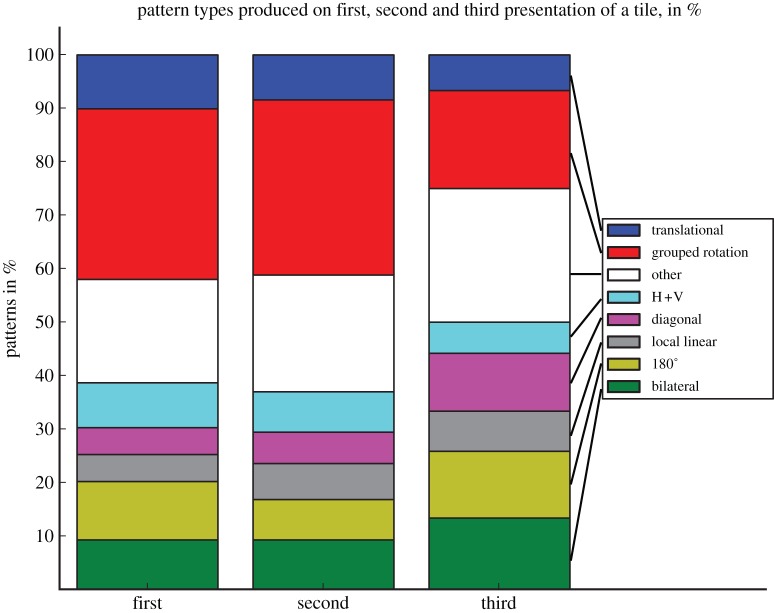
Frequencies of patterns for first, second and third presentations of the tile.

Regarding creativity, participants used the grouped rotation most frequently (31.9% and 32.7% of patterns produced), in the first two passes at a tile. Only with the third pass did grouped rotation frequency drop down to 18.3% (figure 5) and other symmetries, particularly bilateral symmetry along the diagonal axis increase (diagonal symmetry: 6 instances (first iteration), 7 (second iteration), 13 (third iteration)).

Subjects also rated patterns on a Likert scale (1–7, with 7 indicating ‘dislike’). Participants significantly preferred human-made patterns over random patterns, but showed no preference for their own or others’ patterns (table 1; one-way ANOVA: *p* < 0.001, *F* = 30.182, d.f. = 2).

**Table 1.** Mean ratings on a Likert scale (1, ‘I like it’; 7, ‘I do not like it’) for random patterns, other- and own-produced patterns.

image type	mean	*n*	s.d.
unknown (symmetrical)	2.81	54	1.97
unknown (random)	5.00	60	2.17
own	2.77	114	1.69

### (e) Discussion

Our results confirm that humans spontaneously produce highly ordered visual patterns in the absence of instructions to do so. Our participants were also creative, producing many different patterns, and producing different patterns when exposed to the same element repeatedly. Those patterns that do not adhere to classical symmetries still tend to display a high degree of order and organization (see examples in figure 2, images 7*a*–*c*). The local symmetry present in the tile element had an effect on the type of patterns produced (figure 4). Our rates of symmetrical pattern production are somewhat lower than reported by reference [2] (72% versus their 85%). However, those authors used only one type of pattern element (a black dot on a white square), whereas we used three different tile types, which may have promoted the production of a wider variety of symmetries.

This experiment confirms that humans spontaneously impose order on visual arrays, using various generative rules. However, it remains unclear what kinds of structural rules underlying such ordered visual arrays can be perceived. We addressed this question in a series of perceptual experiments, using a ‘spot the flaw’ paradigm described below.

## 5. Experiment 2. ‘spot the flaw’: detection of pattern violations by undergraduates

In the next set of experiments, we studied the perception of plane patterns. We exposed participants to ordered visual patterns, giving them no verbal information about the underlying structural rules. We reasoned that if participants were able to detect violations of the underlying order, or ‘flaws’, then they must have developed some understanding of the regularity of the pattern, even if imperfect. Moreover, we hypothesized that if a pattern type was difficult to perceive or process, aberrations in patterns of that type would be correspondingly more difficult to detect.

### (a) Participants

Sixteen University of St Andrews undergraduates (12 female, mean age: 21.8 years, age range: 18–35) took part in this experiment. All gave their written informed consent prior to participating and were paid for their participation. Participants attested to normal, or corrected to normal, visual acuity, as well as normal colour vision, as a condition of participation. This experiment and experiments 3–5 were approved by the ethics board of the School of Psychology, University of St Andrews.

### (b) Materials

**Figure 6.**
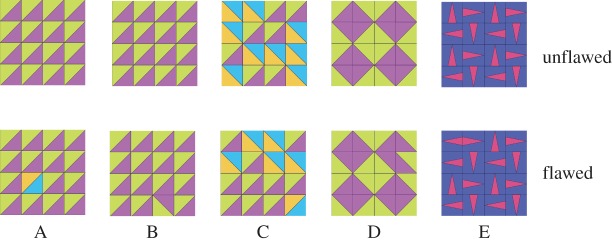
Examples of stimuli used in the five sessions of experiment 2. (A) Colour feature target, (B) orientation feature target, (C) conjunction of colour and orientation features, (D) grouped rotation with tiles that have symmetry on one diagonal axis (E) grouped rotation with tiles that have symmetry on the vertical or horizontal axis.

Five sets of images were shown to each participant (figure 6). In all sets, the grid size varied from a 3 × 3 to a 6 × 6 matrix. The tiles measured 120 × 120 pixels, hence the stimulus size varied from 360 × 360 to 720 × 720 pixels. Each image was shown in a flawed and an unflawed version to each participant, leading to a 50/50 distribution for flawed and unflawed stimuli. In the ‘colour’ task (set A; 64 trials) matrices were of uniform coloured tiles, and flaws consisted of one tile that had different colours. In the ‘orientation’ task (set B; 64 trials), all tiles had the same orientation, and flaws consisted of one tile that had a different orientation. Set C (64 trials) was a ‘conjunction’ task: the matrix contained two tiles with different colours and orientations. A flaw consisted of a tile that had the orientation of one tile type, but the colour of the other tile type (figure 6). Because we expected the following ‘grouped rotation’ tasks to be harder than these single feature tasks, but had no expectation about how much harder, we added the conjunction task as a well-studied point of reference [39,40]. In classical visual search, search times for targets defined by conjunctions typically rise with the number of distracters [39].

The final class of stimuli were generated using a ‘grouped rotation’ rule (64 trials). The tiles were arranged in an orderly hierarchical fashion such that the orientations of the tiles resulted in global shapes consisting of four tiles in the matrix. We split this class into two sets based on tile symmetry: with ‘diagonal symmetry’ tiles (set D; 32 trials) well-formed Gestalt groups were formed. In contrast, the ‘non-diagonal’ tile that made up set E (32 trials) had either a horizontal or vertical symmetry, but no diagonal symmetry, rendering the global shapes less coherent than set D.

The basic tile elements were generated in Inkscape (www.inkscape.org), and then assembled into matrices with Python software implemented in Nodebox (www.nodebox.net). Images were displayed on an LCD monitor; presentation and data collection used custom experiment running software written in Python (Experimenter 1.11). Participants responded via an Iolab button box (www.iolab.co.uk), allowing millisecond reaction time (RT) accuracy.

### (c) Methods

The participants underwent a short interactive training session, during which verbal feedback was given, and all pattern types were shown once. The tiles differed from those used in the test phase. In the test phase, no feedback was given to the participants to indicate whether their decisions were correct or incorrect.

Pilot data showed that class E was more difficult, particularly if combined with images of class D in a single session. Then, class E images were likely to be erroneously classified as flawed, probably owing to the absence of continuation cues between the tiles. Hence, we separated these two image types into two sessions, to avoid confusion as to the cues for the presence of flaws in the images. The order of the sets A–D was randomized for each participant except that set E was always shown last to all participants. Each participant was shown both the flawed and unflawed versions of each stimulus, and the order of the stimuli was randomized within a session.

A two-alternative forced-choice procedure was used. In each test trial, one image was shown on a computer monitor, and participants pressed one of two buttons on the button box to indicate whether or not the presented image contained a flaw (e.g. if an image contained a flaw, the participant had to press the left button, whereas unflawed images required a right button press). Button assignment was constant for each participant, but counterbalanced across participants. A black screen was presented for 1 s between trials. If the participant did not press a button within 10 s, then the trial timed out and the image disappeared from the monitor. Trials that timed out were not repeated and were excluded from the analysis. Participants had an opportunity to take a short break after every 50 trials, as well as between image sets. Response and RT were recorded. Trials with RTs less than 200 ms were excluded, as such short RTs were typically due to inadvertent button presses. Statistical analyses were conducted in SPSS 17.

### (d) Results

Participants performed at very high levels (correct responses for A: 98%, B: 98%, C: 87%, D: 88%, E: 86%); however, the difficulty of tasks C, D and E was reflected in longer RTs (mean RTs in milliseconds: A: 877, B: 970, C: 3110, D: 2227, E: 4318; figure 7). To test whether RTs were different between sessions, we calculated the mean RTs for each session and participant. A Kolmogorov–Smirnov test confirmed that the means were normally distributed for all tasks (all *Z* > 0.6, all *p* > 0.278). Using a repeated-measures ANOVA with a Greenhouse–Geisser correction, the mean RTs differed between tasks (*p* < 0.001, d.f. = 2.568, *F* = 125.57). Pairwise post hoc comparisons showed that tasks A and B did not differ (*p* > 0.99), but that all other tasks differed significantly from each other (*p* < 0.001).

**Figure 7.**
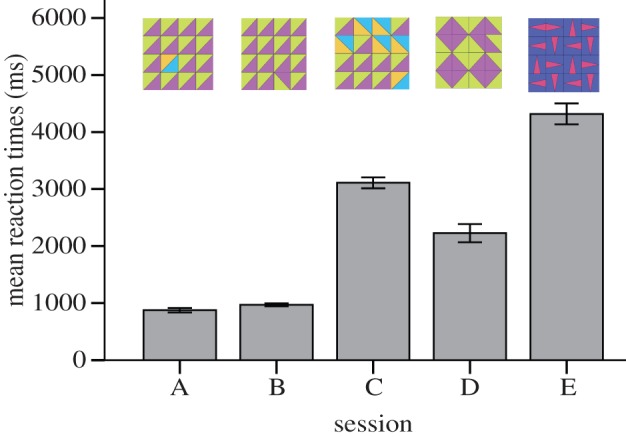
Mean reaction times (milliseconds) for the five tasks in experiment 2. (A) Colour flaw, (B) orientation flaw, (C) conjunction, (D) grouped rotation with tiles that were symmetrical on one diagonal axis, (E) grouped rotation with tiles with vertical/horizontal symmetry. Error bars represent 95% CIs.

### (e) Discussion

These results demonstrate that adults can easily recognize patterns of various sorts, and correctly identify violations of these patterns. Very simple ‘popout’ tasks were trivial, and had high accuracy (98%) and fast reactions. RTs and accuracies for the novel grouped rotation tasks (D and E) were comparable to those in our conjunction task (C). The differences between classes D and E are striking—the presence or absence of continuation or Gestalt grouping cues has a strong effect on RTs (class E responses take about 2 s longer than class D images), but the overall accuracy remains at the same level (88% (D) versus 86% (E)).

## 6. Experiment 3. ‘spot the flaw’: detection of pattern violations by children

Experiment 2 showed that violations in ordered patterns were easily detected by adults. To determine at what age this skill sets in, we conducted an experiment in the same paradigm with children aged 5–12 years. We focused on the two rotation-based tasks from the previous experiment—a simple translational pattern and the hierarchical grouped rotation pattern. We used the more naturalistic tile elements of experiment 1 for more attractive and interesting stimuli.

### (a) Participants

Eighteen children (seven female, mean age 8.76, age range 5–12) and 18 additional university undergraduates (16 female, mean age 21.11, age range 18–35) were tested using a ‘spot the flaw’ task. The undergraduates gave their written informed consent and were paid for participating. A parent or guardian of the child gave their written informed consent, and the children gave their verbal consent and received a small toy for participating.

### (b) Materials

As in experiment 1, digital reproductions of Spanish, Cuban and Portuguese tiles were used. Five different tile images were used. Tiles were repeated on four sizes of square matrix ranging from 3 × 3 to 6 × 6, in both a translational or a rotational pattern (corresponding to task A and D in experiment 2). For each of these 40 images, a corresponding flawed version was created, in which one tile was rotated an additional 90°, yielding 80 stimuli in total. Only tile images with an internal symmetry along one diagonal axis were used, yielding well-formed Gestalt groups. As in previous experiments, each participant saw all stimuli.

### (c) Methods

The experimental session began with a short practice session consisting of 6 stimuli made from different tiles than in the main experiment, but which followed the rules and violations to be tested. Participants received verbal feedback from the experimenter as to whether they had pressed the correct button during the practice session, but not during testing. Images were shown one at a time, and the participants again had to indicate whether or not an image contained a flaw by pressing one of two buttons on a button box. The assignment of buttons was counterbalanced between participants. The participants had to make a decision within 7 s, or the image went away and the screen went black for 1 s. After participant response, the image disappeared and the screen went black for one second. Time-out trials were not repeated, and were excluded from analysis. The participants could optionally take a break after 40 trials.

The experiment used custom-written Python software running on an Apple Mac Mini with an Iolab button box to record responses.

### (d) Results

**Figure 8.**
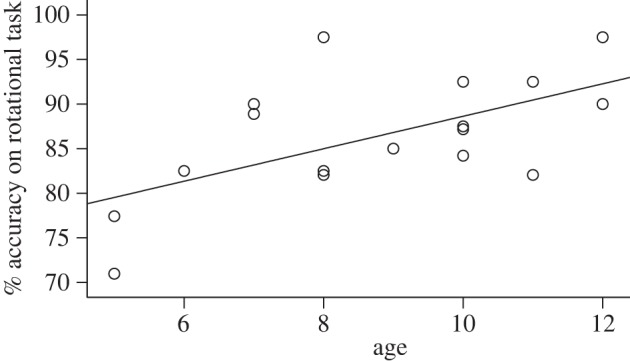
Correlation of percentages of correct responses in grouped rotational patterns and age in children (*r*^2^ linear: 0.358).

Both adults and children performed at high levels of accuracy for both translational and grouped rotational patterns. Adults had 92 per cent correct responses for rotational and 89 per cent for translational patterns, whereas children had 87 per cent correct for rotational and 83 per cent correct for translational patterns. Children's performance was positively correlated with age in the case of rotational patterns (Kendall's Tau-b: *p* = 0.026, *r*^2^ = 0.358; figure 8), but not for translational patterns (Kendall's Tau-b: *p* = 0.124, *r*^2^ = 0.2). No age/performance correlation was found in the adults.

### (e) Discussion

We found that even young children were able to perceive both types of pattern organization without difficulty. Performance when detecting flaws in the simplest rule, translational symmetry, showed no significant improvement with age, whereas the hierarchical grouped rotation rule did. Naturally, it would be intriguing to run a pattern production study such as experiment 1 with children of various age groups to see whether production shows similar development during ontogeny.

## 7. Experiment 4. ‘spot the flaw’: detection of pattern violations in individuals with autism spectrum disorder

Individuals diagnosed with ASD often perform differently in visual tasks [41–43]. Various theories have proposed different processing styles or mechanisms as explanations. The theory of Weak Central Coherence [44–46] maintains that information processing in the visual domain is fundamentally different in individuals with ASD, because the local information is not integrated to a global whole in the same way as in normal individuals, leading to superior performance in tasks that demand a focus on local features, such as embedded figures. Another theory [47–49] posits that a local bias underlies the visual perception in individuals with ASD leading to superior performance in certain visual tasks because there is less distraction from the global information of the stimuli presented. According to this theory, sometimes termed ‘enhanced discrimination’, global perception is intact, though not as dominant as in normal individuals. Not all studies have consistently shown visual search superiority effect in ASD [50], and in fact some have found diminished performance [51]. A recent experiment reports increased sensitivity detecting displays with mirror symmetry in autism compared with typical individuals, which the authors interpret as an ability to access local and global information in parallel [52]. This variety of results and interpretations may stem from high variability between individuals diagnosed with ASD, and suggests that the differences in performance are probably due to multiple factors, rather than any one single factor.

In the next experiment, we compared performance on the ‘spot the flaw’ task used in experiment 2 between ASD individuals and age and IQ-matched controls.

### (a) Methods

Materials and methods were identical to those described in experiment 2.

### (b) Participants

Ten adults diagnosed with ASD (three female, mean age: 22.90, age range: 20–33) and 11 neurotypical adults from the local community (eight female, mean age: 25.36, age range: 20–33) participated in this experiment. All participants, and their guardians if appropriate, gave their informed consent. Participants were paid for their participation. Mean IQ score (as determined by Wechsler Abbreviated Scale of Intelligence test) for the ASD group was 90.8 (s.d. : 19.12) and 101.4 (s.d. : 12.68) for the control group. No significant differences were found between the groups for IQ scores (Mann–Whitney *U*-test: *p* = 0.148, *Z* = −1.446) or age (Mann–Whitney *U*-test: *p* = 0.267, *Z* = −1.109). Furthermore, the differences in IQ scores between males and females were not statistically significant (Mann–Whitney *U*-test: *p* = 0.972, *Z* = −0.35).

### (c) Results

**Figure 9.**
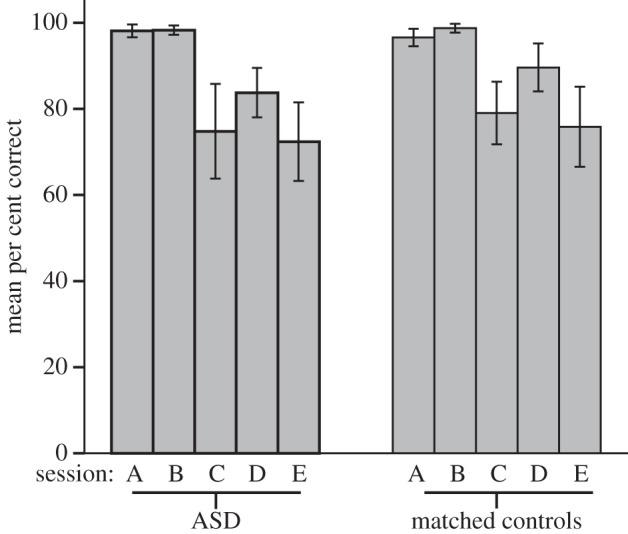
Mean correct responses in per cent for autism spectrum disorder and matched control participants for five flaw detection tasks. (A) Colour flaw, (B) orientation flaw, (C) conjunction, (D) grouped rotation with tiles that were symmetrical on one diagonal axis, (E) grouped rotation with tiles with vertical/horizontal symmetry. Error bars represent 95% CIs.

Both groups mastered the tasks with no problems and intuitively understood what counted as a ‘flaw’ in the pattern. An overview of the mean percentages of correct responses and mean RTs for both groups is shown in figure 9, broken down by stimulus type.

We calculated the mean percentage of correct response for both groups in the different sessions. These values were normally distributed. (Kolmogorov–Smirnov tests, all *p* > 0.169, all *Z* > 0.755.) A mixed-model ANOVA with *stimulus type* (i.e. sessions A–E) as a within subjects factor and *group* as a between subjects factor showed that per cent correct for the different tasks did not differ significantly between the groups (test of within subject effects for session and group affiliation with Greenhouse–Geisser correction: *p* = 0.483, *F* = 0.784, d.f. = 2.380). Mean RTs for each task and participant were normally distributed for all tasks (Kolmogorov–Smirnov, all *p* > 0.223, all *Z* > 0.571). A mixed-model ANOVA showed no significant differences between the groups for the mean RTs for each session (within subject effects for session and group affiliation with Greenhouse–Geisser correction: *p* = 0.352, *F* = 1.068, d.f. = 1.914).

Pairwise comparisons of mean correct responses for each individual group showed significant differences in accuracy between the tasks within groups (repeated measures ANOVA with Greenhouse–Geisser correction, ASD: *p* < 0.001, *F* = 22.843, d.f. = 2.329; controls: *p* < 0.001, *F* = 21.762, d.f. = 1.995). Session C and E did not differ significantly from each other for either group (ASD: *p* = 0.586, controls: *p* = 0.225), suggesting that the conjunction task and grouped rotation with good Gestalt figures were similarly challenging for both groups. RTs also showed significant differences between the sessions (within subject effect, Greenhouse–Geisser correction: ASD: *p* = 0.001, *F* = 14.707, d.f. = 1.461; controls: *p* < 0.001, *F* = 63.16, d.f. = 2.43; figure 9). The mean RTs for session E were significantly higher than all other tasks in the control group (all pairwise *p* < 0.04). For the ASD group, the mean RT for session E differed from all sessions (all *p* < 0.05) except session C, the conjunction task (*p* = 0.845).

### (d) Discussion

In contrast to previous research on visual search in ASD individuals, we did not find consistently higher rates of accuracy or faster RTs for our ASD group. The control group did show slower RTs in task E than in the other tasks, whereas the ASD group did not show such an effect. Yet, because the percentage of correct responses did not differ between tasks C and E for either group, the apparent speed advantage that the ASD group showed for this, the most difficult task, did not coincide with higher accuracy. However, given that correct responses were all well above chance level, it is not surprising that this possible slight advantage for the ASD group is reflected in RTs rather than differences in response accuracy. Overall, this data reveal no appreciable differences between ASD and control groups in solving our tasks presented to them.

## 8. Experiment 5. serial versus hierarchical rotation

### (a) Introduction

Experiments 2–4 showed that human participants readily detect violations of rule-based patterns. In this experiment, we initiated a more systematic investigation of the specific types of patterns that can be more or less easily parsed. We used two minimally different production rules. The first implements the most frequently produced pattern in experiment 1 (‘grouped rotation’). This hierarchical pattern, made up of subunits of four tiles, is not affected by changes in grid size, because the four orientations are relative to a fixed point, which leads to mid-level visual groupings of four tiles. If the matrix size is odd, then these groupings are incomplete on the edge, but the overall pattern remains unchanged. The production rule underlying the second, ‘sequential’ pattern entails that the tiles are rotated by 90° when serially progressing along the grid in a Western reading fashion (from top left to bottom right), with no fixed point around which the rotation occurs. Despite their simplicity, patterns with serial rotation were never produced spontaneously in experiment 1. Unlike the grouped rotation, the pattern that is produced with a serial rotation depends strongly on the overall matrix size. Obviously, we cannot know whether the patterns are necessarily perceived using these rules. Indeed, we cannot, at present, speculate about what the rules underlying the parsing of patterns by humans are. However, the production rules explicitly describe how the patterns were generated computationally.

### (b) Materials

**Figure 10.**
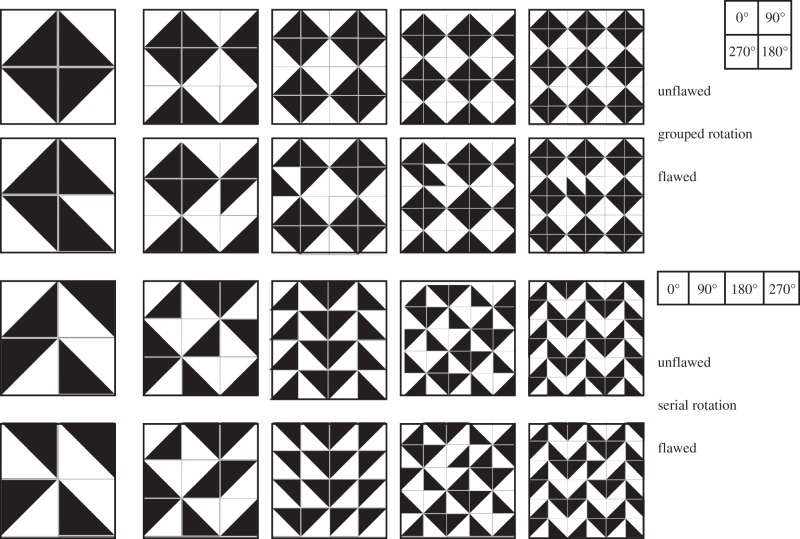
Examples of stimuli generated in matrices ranging from 2 × 2 to 6 × 6, in hierarchical grouped and serial rotations, and in flawed and unflawed versions (recreated in black and white).

We used 13 images of Spanish and Portuguese tiles from the same sources as experiment 3 and arranged them on five matrix sizes, ranging from 2 × 2 to 6 × 6, with custom-written Nodebox software according to both the hierarchical and the sequential rules. Each image was generated in a flawed and unflawed version, yielding a total of 260 images. The tiles were 120 × 120 pixels, hence the stimulus size varied from 240 × 240 to 720 × 720 pixels. As the matrix size increases, the composite structures that emerge in patterns using the serial rotation rule are very different: 4 × 4 and 6 × 6 matrices contain structures that repeat along the vertical and horizontal axes, whereas 3 × 3 and 5 × 5 matrices contain structures that repeat along the diagonals (see figure 10 for examples). Generally, structures on a diagonal are often more difficult to perceive (oblique effect), most likely due to a diminished neural representation compared with horizontal and vertical structures [53].

### (c) Participants

Twelve university undergraduates (nine female, mean age: 20.9, age range: 21–40) took part in this study. They gave their written informed consent prior to the experiment and were paid for their participation.

### (d) Methods

The order of all images was randomized. Images were shown one by one on a computer monitor, and the participants had to indicate by pressing one of two mouse buttons whether they found a flaw in the image or not. The assignment of mouse buttons to flawed or unflawed images was counterbalanced across participants. There was no time limit for responding. The software was run with custom-written Python software on an Apple Mac Mini.

### (e) Results

Trials with RTs shorter than 200 ms were excluded, as these were most likely accidental button presses (nine out of 3120 trials). Across all grid sizes, participants responded much faster and more accurately for hierarchical ‘grouped rotation’ than sequential patterns, with RTs roughly twice as long for images with serial rotation (mean RT for serial rotation: 1.66 s, grouped rotation: 3.21). This difference in mean RTs was significant (Mann–Whitney *U*-test: *p* = 0.013, *U* : 29).

Accuracy was high for hierarchical patterns for all grid sizes (table 2). Grid size affected accuracy in different ways in the two patterns. In hierarchical patterns, participants were significantly better in the 2 × 2 images than all other grid sizes (two-way ANOVA, *F*_1,4_ = 15.48, for *matrix size ×* *pattern*: *p* < 0.001, Tukey pairwise comparisons *p* < 0.001) with the exception of 4 × 4 (*p* = 0.09), whereas in the sequential rotation, participants were significantly better at 4 × 4 images than all other grid sizes (all pairwise comparisons *p* < 0.001). Differences in performance between grid sizes for the sequential patterns do not appear to reflect an oblique effect, as there is no statistical difference between 5 × 5 grids (diagonal repetition) and 6 × 6 grids (vertical repetition), *p* = 0.96. Accuracy was lower in the serial rotation task but still above chance. We computed *d*′-values as a measure of sensitivity to flaws in patterns [54,55]. *D*′-values were higher for all participants on grouped rotation (mean *d*′ for grouped: 3.01, for sequential: 1.03), and in total were significantly higher for this pattern (Wilcoxon-signed rank test: *p* = 0.002, *Z* = −3.059). We conclude that the production preference for hierarchically grouped patterns, observed in experiment 1, is closely mirrored by perceptual performance. Participants found a computationally simple but visually non-intuitive pattern quite difficult to parse but performed above chance, even though they were unlikely to have encountered such patterns earlier.

**Table 2.** Accuracy (in %) for hierarchical and sequential patterns.

pattern	2 × 2	3 × 3	4 × 4	5 × 5	6 × 6
hierarchical	98.4	87.8	92.6	85.9	87.5
sequential	59.5	65.9	88.4	62.1	66.2

### (f) Discussion

Results from experiments 2–5 provide strong evidence that humans intuitively understand a concept of orderedness in visual patterns and use it to reliably detect violations in patterns of varying levels of complexity. Whether animals detect such rules in two-dimensional patterns remains unclear. Testing these questions is not easy—the animal in question has to be highly visual (using vision either for foraging or navigation, or both) and trainable in a laboratory setting. We decided to use pigeons as a first species to test on our patterns, as their visual system is well understood and a large body of research shows that they can be excellent visual learners.

## 9. Experiment 6. ‘spot the flaw’: detection of pattern violations in pigeons

### (a) Introduction

Visual perception is well studied in pigeons [56], and it seems clear that in many ways their perception is different from humans. For example, when presented with stimuli that are hierarchically organized, with information available on the local and global level, typical humans give precedence to global-level information rather than local [57]. Studies on pigeons reached conflicting conclusions concerning their global parsing abilities. Cavoto & Cook [58] found that pigeons learned to discriminate between shapes based on local information more readily, suggesting that local information takes precedence in their visual processing. By contrast, Goto *et al.* [59] found that global-feature properties were acquired faster than local features, as in humans.

Rotational violations of translational patterns (see experiment 2, task B) could be detected using only local perception, i.e. spotting the one tile that has a different orientation than any neighbours. However, solving the same task with a hierarchically grouped pattern requires processing beyond the strictly local level, as the ‘flawed’ tile does not have a unique orientation in the array. Thus, recognizing a flaw requires that the orientation of each tile be checked against its neighbours to see whether or not the orientation fits.

In this experiment, we tested orientation flaws in both translational patterns and grouped rotational patterns, and included a simple control task consisting of detecting a colour flaw.

Pigeons outperform humans on tasks that involve judging whether complex geometrical shapes are identical in various rotations [60], but perform worse than humans on tasks that require them to judge whether or not an object contains bilateral symmetry. Delius and co-workers found some evidence for bilateral symmetry recognition in pigeons [61,62], whereas Huber *et al.* [63] did not.

### (b) Subjects

Eight pigeons (*Columba livia*) participated. All birds were socially housed in outdoor aviaries in Vienna. The experiment was conducted in accordance with Austrian animal protection laws; see Huber [64] for animal housing and care procedures. The birds were trained 5 days a week, and had free access to water and grit in their aviaries. On days when experiments were conducted, food was available only during or immediately after the experimental sessions.

### (c) Materials

**Figure 11.**
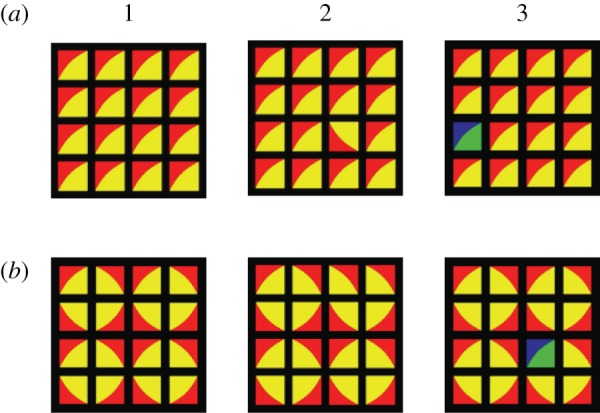
Examples of patterns used in experiment 5. (*a*) Translation and (*b*) grouped rotation: (1) unflawed, (2) with orientation and (3) colour flaws.

We used square 4 × 4 matrices. Four tiles with different internal features and four colours (red, blue, yellow and green) were used. The tiles were divided along the diagonal, with the colour division being roughly equal between the two halves. Each tile consisted of two colours. All possible colour combinations were used in the tiles, and the tiles either had 0° or 90° initial orientations, leading to 96 flawed and unflawed image pairs in total. The stimuli measured 3.7 × 3.7 cm on screen—the tiles were squares of 0.7 × 0.7 cm, with 2.5 mm black borders between the tiles. The tiles were arranged either in a translational or a rotational pattern (figure 11). The stimuli were generated with custom-written Nodebox software.

In the colour control task, the target consisted of one tile that had the correct orientation, but a different colour combination than the other tiles. The target was randomly located in the matrix. For both rotational and translational patterns, the violation was unique in the array only due to its distinctive colour features. In the structure task, the target was one tile which had an orientation that did not follow the pattern, but had the same colours as the rest of the array. Crucially, the violation was unique in the matrix in the translational pattern only. In the rotational pattern, the target was not unique in the array and could only be detected if the relation of the tile to its neighbours was taken into account.

### (d) Apparatus

The pigeons were individually placed in an experimental chamber, equipped with an infrared touch screen (CarrollTouch, 15″). Underneath the touch screen was an opening through which a feeding device provided grain rewards after a correct response. The feeder tray was raised and illuminated for 3 s to provide a food reward. The experiment, including the apparatus, stimulus presentation and data recording was run using the software CogLabLight, v. 1.9 (Michael Steurer).

### (e) Methods

One group of experimentally naive birds (*n* = 4) were trained on a colour task, and one group of experienced birds (*n* = 4) were trained on both the colour and structure targets in a Go/No-Go paradigm (see [65] for an extensive description of the apparatus and paradigm). One image, either flawed or not, was presented at a time. Within each group, half the birds were trained to peck on images with a flaw to get a food reward, whereas the other half were trained to peck on images with no flaw.

The number and timing of the bird's pecks in the first 10 s of stimulus presentation were recorded for analysis. After a varying time interval (VI) of 10–30 s, the pecks were again counted to trigger the reward. In this final phase of the trial, the bird had to peck at least five times, and three times within 3 s on an S+ or ‘Go’ image to get a food reward. If a negative (S−) image was shown, the bird had to withhold pecking for at least 8 s after the VI (No-Go). No reward was given for withholding pecking. Pecking on a No-Go stimulus was not penalized, except that the image did not go away and the trial did not finish until pecking was withheld for the requisite time period. The order of trials was randomized. The criterion for passing the task was five successive sessions with *ρ*-values significantly above chance (*ρ* > 0.726). The *ρ*-value is derived from the *U*-value in a Mann–Whitney test, and is commonly used in categorization experiments [66,67]. The training was aborted if the bird did not reach this criterion after 165 sessions (3960 trials).

In addition to the colour control task, the four experienced birds were also trained to distinguish between images that did or did not contain a structural flaw. All birds were tested on both patterns (two starting with translational and then rotational, and two birds in the reverse order). For half the birds, the flawed image was (S+).

### (f) Results

**Figure 12.**
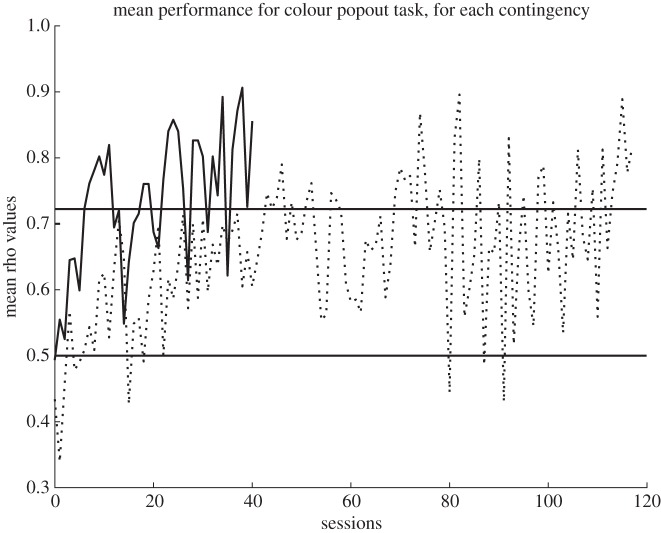
Training progress of pigeons (*n* = 4) on the colour flaw task. Dotted line, flawed image is S−; solid line, flawed image is S+. The upper horizontal line represents the significance threshold (*ρ* = 0.72), whereas the lower line represents chance level (*ρ* = 0.5).

All eight birds mastered the colour task. Both naive and experienced birds showed a feature positive effect [68,69], i.e. those birds trained to peck on images that contained a colour flaw reached criterion significantly faster than birds trained to peck on images that did not contain a flaw (Mann–Whitney *U*-test: *p* = 0.029, *Z* = −2.309; figure 12).

Despite their success in the colour task, none of the birds managed to discriminate reliably between the two image classes in the orientation task regardless of whether the flaw was located in a translational or rotational pattern, or whether it was (S+) or (S−) (see electronic supplementary material). Training with structural flaws failed to reach criterion and was aborted after 165 sessions (3960 trials).

Thus, pigeons learned to detect a colour feature with relative ease, but a task that requires the detection of a structural feature, even a simple one, was not mastered by the birds in this experiment.

### (g) Discussion

These results are surprising because, in principle, the unique structure feature in the translational pattern should be solvable by strictly local processing. The fact that the birds consistently failed the task suggests that rotational invariance may make orientation anomalies hard to detect. However, Cook *et al.* [70] have shown that pigeons can differentiate vertical and horizontal orientations of the same object, and that performance is no worse and acquisition no slower for this orientation cue than for colour or size cues. The stimuli in that study also consisted of identical elements arranged on a square grid, but differed from ours in that the target was not one single element with a different orientation, but a square group of 7 × 6 elements with a different orientation than the rest of a 24 × 16 matrix, thus the orientation targets took up a larger portion of the matrix overall. The stark contrast in performance between the colour and orientation tasks suggests that our pigeons had severe difficulties processing the types of patterns that all groups of humans mastered in the preceding experiments.

## 10. Summary and general discussion

The picture that emerges from this suite of experiments is very clear: humans are excellent at parsing and creating ordered patterns. Patterns spontaneously created in experiment 1 are not only highly ordered, but also show symmetries of various types. Evidently, humans have a strong drive to impose order on random arrays, and do so without instruction. Furthermore, our participants gave ordered patterns higher preference ratings than random patterns, providing empirical support for Gombrich's assertion that humans prefer ordered visual arrays over random ones. In the patterns spontaneously produced, the most frequent pattern (grouped rotation) was observed most often in participants’ first encounter with a new tile, and dropped off when the tile was encountered a third time, suggesting that there is a trade-off between reaching an ‘obvious’ default solution to the self-imposed task of creating ordered arrays and exploring creative, but not necessarily symmetrical, tile arrangements

In our perceptual studies, we contrasted perception of a very simple pattern, translational symmetry, with the most frequent pattern from the pattern production experiment, grouped rotation, by normal adults, individuals diagnosed with ASD and children. All of our human participants intuitively understood what a pattern was and what counted as a violation. Performance for adults was at very high levels, and ASD individuals performed at the same levels of accuracy as age and IQ-matched neurotypical participants.

Finally, approaching the question of pattern perception from a comparative direction, we investigated which violations of patterns pigeons could detect. In sharp contrast to humans, the pigeons tested on structural violations clearly failed at the task, whereas succeeding on a comparable colour task. The universal mastery of the task in humans, contrasted with the pigeons’ failure, suggests that visual patterns tap into cognitive skills that might be phylogenetically unusual: creating and parsing rule-governed structures that can be reiterated indefinitely seems trivially easy to humans. Surprisingly, pigeons—a highly visual bird species—could not master such structures.

Given that language, music and the visual arts are all typical aspects of human cultures worldwide, we might ask to what degree the cognitive resources that underlie these three domains are shared and general, versus domain-specific. A growing body of research suggests that music and language may share processing resources in the brain [71]. When we contrast abstract visual patterns with this pair, two immediate comparisons suggest themselves: hierarchy and symmetry.

Regarding hierarchy, both music and language are typified by hierarchical, tree-like structures, in which small constituents are combined into larger and larger components to create a multipart whole. Although abstract visual patterns do not need to display such hierarchy, we found that humans, left to their own devices, have a strong propensity to generate hierarchical patterns (the ‘grouped rotation’ pattern being the most common), and that perceivers find such patterns easy to process. Furthermore, discrimination over hierarchical patterns improves in children with age, implying that the underlying cognitive processes of these visual patterns develop increasing sophistication with maturity. In general, our results suggest that hierarchy represents an important similarity of decorative visual patterns with musical or linguistic patterns.

By contrast, symmetry seems to be a domain of difference between visual patterns and music or language—at least superficially. Syntactic structures in language tend to be asymmetrical [72], with tree structures branching either to the right or left depending on the language. By contrast, both artisanal decoration and the patterns generated in our experiments tend to show one or more axes of symmetry. However, this apparent dissimilarity may be an artefact of linearization in the acoustic domains, i.e. reducing dimensionality from a multi-dimensional (and more symmetrical) conceptual space down to a one-dimensional auditory/vocal stream. Visual patterns extending in a two-dimensional plane are not similarly constrained—unlike language, their output entails no inherent structural asymmetry. If visual pattern perception relies upon processing resources that overlap with those of language and/or music, then the greater dimensional freedom of two-dimensional patterns may offer new insights into types of dependencies and symmetries that can be processed by these general perceptual mechanisms, including types that by definition cannot occur in music or language, such as multi-dimensional long-distance dependencies. We believe that applying the theoretical framework of formal language theory to two-dimensional patterns offers a rich new perspective on the human capacity for producing regular, hierarchically organized structures. Such visual patterns may actually prove more flexible than music or language for probing the full extent of human pattern processing abilities.

With the results presented here, we have taken the first steps in decoding the uniquely human fascination with visual patterns, what Gombrich termed our ‘sense of order’.

Although the patterns we studied are most similar to tilings or mosaics, they are examples of a much broader type of abstract plane pattern, a type found in virtually all of the world's cultures [4]. Given that such abstract visual patterns seem to represent human universals, they have received astonishingly little attention from psychologists. This neglect is particularly unfortunate given their democratic nature, their popular appeal and the ease with which they can be generated and analysed in the laboratory. With the current research, we hope to spark renewed scientific interest in these ‘unregarded arts’, which we believe have much to teach us about the nature of the human mind.
